# Health Care Needs and Costs for Children Exposed to Prenatal Substance Use to Adulthood

**DOI:** 10.1001/jamapediatrics.2024.2281

**Published:** 2024-07-22

**Authors:** Evelyn Lee, Deborah Schofield, Mithilesh Dronavalli, Kate Lawler, Hannah Uebel, Lucinda Burns, Barbara Bajuk, Andrew Page, Yuanyuan Gu, John Eastwood, Michelle Dickson, Charles Green, Lauren Dicair, Ju Lee Oei

**Affiliations:** 1 Centre for Social Research in Health, University of New South Wales, Kensington, New South Wales, Australia; 2 Centre for Economic Impacts of Genomic Medicine, Macquarie Business School, Macquarie University, North Ryde, New South Wales, Australia; 3 Translational Health Research Institute, Western Sydney University, Penrith, New South Wales, Australia; 4 Discipline of Paediatrics and Child Health, School of Clinical Medicine, University of New South Wales, Australia; 5 Department of Paediatrics, Sydney Children’s Hospital, Sydney, New South Wales, Australia; 6 Macquarie University Centre for the Health Economy, Macquarie Business School and Australian Institute of Health Innovation, Macquarie University, North Ryde, New South Wales, Australia; 7 National Drug and Alcohol Research Centre, University of New South Wales, Kensington, New South Wales, Australia; 8 Critical Care Program, Sydney Children’s Hospitals Network, Sydney, New South Wales, Australia; 9 National Public Health Service, Te Whatu Ora – Health New Zealand, Dunedin, New Zealand; 10 School of Population Health, University of New South Wales, Kensington, New South Wales, Australia; 11 Department of Preventative and Social Medicine, University of Otago, Dunedin, New Zealand; 12 Sydney Institute for Women Children and their Families, Sydney Local Health District, Sydney, New South Wales, Australia; 13 Menzies Centre for Health Policy and Economics, School of Public Health, University of Sydney, Camperdown, New South Wales, Australia; 14 Early Years Research Group, Ingham Institute for Applied Medical Research, Liverpool, New South Wales, Australia; 15 The Poche Centre for Indigenous Health, Faculty of Medicine and Health, The University of Sydney, Camperdown, New South Wales, Australia; 16 Alpha Maxx Healthcare, Memphis, Tennessee; 17 Private Practice, Havertown, Pennsylvania; 18 Department of Newborn Care, Royal Hospital for Women, Randwick, New South Wales, Australia; 19 Drug and Alcohol Services, Murrumbidgee Local Health District, Wagga Wagga, New South Wales, Australia

## Abstract

**Importance:**

Children exposed to substance use during pregnancy have increased health needs but whether these are influenced by engagement in out-of-home care is uncertain.

**Objective:**

To evaluate the association between substance use during pregnancy, out-of-home care and hospitalization utilization, and costs from birth up to age 20 years.

**Design, Setting, and Participants:**

This was a retrospective cohort study using individual-linked population birth, hospital, and out-of-home care information of all liveborn infants from New South Wales, Australia, between 2001 and 2020 using longitudinal population-based linkage records from administrative databases. Substance use during pregnancy included newborns with neonatal abstinence syndrome (n = 5946) and intrauterine exposure to drugs of addiction (n = 1260) and other substances (eg, tobacco, alcohol, and illicit drugs or misused prescription drugs; n = 202 098). Children not exposed to substance use during pregnancy were those without known exposure to substance use during pregnancy (n = 1 611 351). Data were analyzed from July 2001 to December 2021.

**Main Outcomes:**

Main outcomes were hospital readmission, length of stay, and cost burden associated with substance use during pregnancy from birth up to age 20 years. Outcomes were investigated using 2-part and Poisson regression models adjusted for sociodemographic characteristics. Mediation analysis was used to evaluate whether the association of substance use during pregnancy with risk of readmission was mediated through engagement with out-of-home care.

**Results:**

Of the 1 820 655 live births, 935 807 (51.4%) were male. The mean (SD) age of mothers was 30.8 (5.5) years. Compared with children who were not exposed to substance use during pregnancy, those who were exposed incurred significantly higher birth hospital costs (adjusted mean difference, A$1585 per child [US$1 = A$1.51]; 95% CI, 1585-1586). If discharged alive, more children with exposure to substance use during pregnancy had at least 1 readmission (90 433/209 304 [43.4%] vs 616 425/1 611 351[38.3%]; adjusted relative risk [RR], 1.06; 95% CI, 1.06-1.07), most commonly for respiratory conditions (RR, 1.11; 95% CI, 1.09-1.12) and mental health/behavioral disorders (RR, 1.36; 95% CI, 1.33-1.41). Excess hospital costs associated with substance use during pregnancy were A$129.0 million in 2019 to 2020. Mediation analyses showed that any out-of-home care contact mediated the association between substance use during pregnancy and risk of inpatient readmission and lower health care cost (decreased by A$25.4 million). For children with neonatal abstinence syndrome, any out-of-home care contact mediated readmission risk by approximately 30%, from adjusted RR, 1.28; 95% CI, 1.19-1.35, to RR, 1.01; 95% CI, 0.98-1.02.

**Conclusion and Relevance:**

Children who were exposed to substance use during pregnancy incurred more hospital costs than children who were not exposed up to 20 years of age, but this was reduced in association with any contact with out-of-home care. This provides insights into possible strategies for reducing health and financial burdens associated with exposure to substance use during pregnancy for children.

## Key Points

**Question:**

Does the increase in health care needs among children exposed to substance use during pregnancy vary by engagement in out-of-home care?

**Findings:**

In this cohort study, children exposed to substance use during pregnancy with or without neonatal abstinence syndrome were at higher risk of adverse birth outcomes and long-term costs than children who were not exposed but a reduction in cost was associated with any out-of-home care contact.

**Meaning:**

Increased support and timely access to services could mitigate the higher readmission risk and cost associated with substance use during pregnancy.

## Teaser

This cohort study assesses associations among substance use during pregnancy, health care needs among children, and out-of-home care.

## Introduction

Health care for children exposed to maternal substance use during pregnancy constitutes an enormous burden on health services^1,2^ In the US, costs associated with neonatal abstinence syndrome (NAS; also called newborn withdrawal) have tripled in 7 years, with direct costs for birth admissions increasing from $731.8 million in 2009 to $2.5 billion in 2016 (or $79 937 per infant).^1^ In the UK, NAS was estimated to cost the health system £62.6 million ($76.4 million) per year or £9771 per infant ($11 930).^3^

After hospital discharge, children with NAS have consistently higher rates of rehospitalization. We, and others,^4,5,6^ have shown that NAS is associated with rehospitalization for preventable conditions such as injury and maltreatment, as well as for mental and behavioral health disorders.^4^ Collectively, this translates into higher health care costs for children with NAS until early childhood.^5,6,7^

Not all children with exposure to substance use during pregnancy develop NAS but there is little information on those without NAS, especially those with nonopioid-related NAS.^8,9,10^ In addition, due to difficulties in tracking large numbers of vulnerable children for long periods, little is known about lifestyle and environmental impact on the health needs of children with exposure to substance use during pregnancy. Indeed, in Australia, up to 50% of children with NAS are placed in out-of-home care by age 5 years,^11^ but how this impacts their health outcomes is unknown.

The need to evaluate the mediating effect of any contact with out-of-home care and substance use during pregnancy is important because both problems increase health care costs. A previous Australian study showed that individuals who had experienced misuse and neglect in childhood incurred more than A$124 million (US$1 = A$1.51) on public hospital costs from birth to age 31 years, resulting in A$3.8 billion excess hospital costs.^12^ Children in out-of-home care are especially at risk of poor mental health^13^ and other conditions, such as dental problems and developmental delay,^14,15^ and have been shown to have difficulties accessing necessary and even routine health care.^16^

In this study, we used individual-linked administrative population data to examine rates of readmission, length of stay (LOS), reasons for and time to readmission, and health care costs from birth to a maximum of 20 years of age for children with intrauterine exposure to drugs of addiction, including tobacco, alcohol, illicit drugs, or misused prescription drugs, with and without NAS. We compared their outcomes with those of children in the population without known exposure to substance use during pregnancy.

We hypothesized that children exposed to substance use during pregnancy regardless of a diagnosis of NAS would have more hospital readmissions and incur greater costs at birth and into young adulthood compared with children with no known substance exposure, but that any contact with out-of-home care would mediate the association between substance use during pregnancy and hospital readmission and related costs.

## Methods

This study used longitudinal population-based linkage records from administrative databases (eTable 1 in Supplement 1). The primary database was the Perinatal Data Collection, from which we collected maternal and infant information for births between July 1, 2001, and December 31, 2020, in New South Wales (NSW), the most populous state in Australia. Ethics approval was obtained from the NSW Human Research Ethics Committee, the Australian Capital Territory, and the Aboriginal Health and Medical Research Council. We followed the Strengthening the Reporting of Observational Studies in Epidemiology (STROBE) Strengthening the Reporting of Observational Studies in Epidemiology (STROBE) reporting guideline for cohort studies.

Data linkage was conducted by the Centre for Health Record Linkage, which linked the Perinatal Data Collection to other population datasets in NSW. The validity of probabilistic record linkage was high between maternal hospital and birth records (98.1%).^17^

In our study, substance use during pregnancy was defined according to *International Statistical Classification of Diseases and Health Related Problems, Tenth Revision, Australian Modification* (*ICD-10-AM*) codes, using 51 diagnostic fields relevant to any infant up to 20 years after birth and, for mothers, 2 years before the birth of the child. The Australian Refined Diagnosis Related Groups (AR-DRGs) code reflects the resources required by the hospital was used to estimate costs. Study Exposures (eFigure 1 in Supplement 1)

All live-born infants (N = 1 820 655) after excluding stillbirths (n = 11 188) and infants with missing discharge data for the birth admission (n = 2842) were categorized into 7 mutually exclusive groups. The substance use during pregnancy classification was based on newborn birth records as well as maternal data on type of substance use (*ICD-10-AM* F10-F19: mental or behavioral disorders due to psychoactive substance misuse) recorded in the mother’s last hospital admission or an outpatient mental health care episode within 2 years before the birth. Children exposed to substance use during pregnancy were compared with children with no known history of prenatal substance exposure either from infant or maternal records (n = 1 611 351).

Children with prenatal substance exposure (n = 209 304) were categorized into 6 mutually exclusive groups: (1) those exposed to only maternal smoking (*ICD-10-AM* F17.2; n = 186 485), (2) those exposed to only maternal alcohol misuse/dependence (*ICD-10-AM* F10.1-10.2, F10.9, and P04.3; n = 2781), (3) those exposed to both maternal smoking and alcohol misuse/dependence (*ICD-10-AM* F17.2 and F10.1-10.2, F10.9, or P04.3; n = 1866), (4) those exposed to maternal mental or behavioral disorders due to psychoactive substance misuse but no newborn birth records of a prenatal substance exposure diagnosis (*ICD-10-AM* P04.4) or NAS diagnosis (*ICD-10-AM* P96.1) (n = 10 966), (5) those diagnosed with prenatal substance exposure (*ICD-10-AM* P04.4; n = 1260), and (6) those diagnosed with NAS (*ICD-10-AM* P96.1; n = 5946).

### Out-of-Home Care

This study investigated the indirect association of any out-of-home care contact with the risk of inpatient readmission in children with substance use exposure during pregnancy, adjusted for confounders. Information on out-of-home care was derived from administrative records held by the NSW Department of Communities and Justice (DCJ). Information about the characteristics of children and placement histories—such as demographic characteristics, child protection history, and types of placements—were obtained. In Australia, out-of-home care refers to court-ordered placement of the child at any age younger than 18 years in alternative care arrangements, such as foster care, relative/kinship care, adoption, and residential care, for any period of time due to risk to safety in their biological home. Out-of-home care is thus an indicator of substantiated child harm, of engagement with child protection services, and of the need for increased surveillance and support. Variables were created for children with any episode of out-of-home care placement (yes/no), kinship care (yes/no), and a continuous variable related to the mean age (in years) in their initial out-of-home care placement.

### Hospital Utilization and Costs

The risk of readmission was defined as any inpatient hospital admission after birth discharge. Hospitalization costs were assigned to each episode using the AR-DRGs–specific mean national public hospital costs, adjusted to 2019 to 2020 Australian dollars and by type of substance use during pregnancy.^18,19,20^ Cost results were extrapolated to the average total live births in Australia each year to determine the national annual health cost burden, from a health care perspective.

The study used directed acyclic graphs to identify and adjust for potential confounders that are known to be associated with substance use during pregnancy and the study outcomes from the literature including maternal age younger than 20 years, mothers who identified as Aboriginal and/or Torres Strait Islander, socioeconomic status decile (based on the Index of Relative Socio-economic Advantage/Disadvantage),^21^ maternal psychosocial factors including serious mental health disorder (yes/no), and calendar year^22,23^ (eFigure 2 in Supplement 1).

### Statistical Analysis

Poisson regression was used to calculate the relative risk (RR) with 95% CIs. Given the high proportion of zero costs, as some individuals had no inpatient readmission visits for each period of interest (after the birth admission), the classical 2-part model approach was used to assess the health care costs. The first part was a multivariate logistic regression model predicting the probability of inpatient readmission. The second part modeled the distribution of costs conditioned on having incurred positive costs (for each period of interest) using a generalized linear model with a γ distribution and a log link. The 2 parts are assumed to be independent and estimated separately.^24^ Robust standard errors were used to account for possible violations of the error term (Huber-White sandwich estimators).

Causal mediation analyses using the mediate command in Stata version 18 (StataCorp) were conducted to investigate the study question as to what the risk among children with substance use exposure during pregnancy would be if they had not experienced out-of-home care after adjusting for potential confounders.^25^ Causal mediation analyses use a counterfactual approach that assesses the mediation by out-of-home care in the risk of exposure to substance use during pregnancy on readmission and thus circumvents the problem of collider bias^26^ commonly found in traditional approaches that use conditional models to investigate mediation.^27^ In ascribing inverse probability weights to individuals in the dataset, the indirect effect in the mediation analysis essentially recodes those children exposed to prenatal substances children and out-of-home care to have the same characteristics of those who did not experience out-of-home care.

Specifically, causal mediation analyses estimate the causal effect of substance use during pregnancy on the risk of readmission (outcome)—that is, the total effect, which can be divided into (1) natural direct effect of exposure to substance use during pregnancy on the outcome and (2) natural indirect effect of exposure to substance use during pregnancy on the outcome via the mediator (ie, any out-of-home care contact). The natural indirect effect is the observed effect of substance use during pregnancy on the risk of readmission if all children with exposure to substance use during pregnancy were placed in out-of-home care, whereas the natural direct effect assessed the risk of readmission in the absence of any out-of-home care contact in the cohort (ie, the expected risk of readmission if a child was not placed in out-of-home care). The costs associated with readmission by type of maternal substance use were assessed at the patient level as the product of the adjusted mean cost of readmission for each period of interest and the excess relative risk of readmission from the mediating analyses. The reasons for readmission through to young adulthood were based on the diagnosis codes recorded in any of the diagnosis fields for each hospital admission using the validated list of *ICD-10-AM* codes.

All analyses were performed using Stata version 18 (Stata Corp). Data were analyzed from July 2001 to December 2021.

## Results

### Population Characteristics

This study included 1 820 655 live births (935 807 [51.4%] male; 884 848 [48.6%] female; mean [SD] age of mothers, 30.8 [5.5] years). Of these, 209 304 infants had prenatal substance exposure (114.9 per 1000 live-births), including 5946 diagnosed with NAS as infants (3.26 per 1000 live-births). At birth, 21 948 neonates (10.5%) with exposure to substance use during pregnancy were premature (<37 weeks’ gestation), 23 787 (11.4%) had low birth weight (<2500 g), and 5136 (2.5%) were admitted to a special care unit or a neonatal intensive care unit; 812 (0.4%) infants with exposure to substance use during pregnancy died prior to hospital discharge compared with 3689 infants (0.2%) who were unexposed. Mothers of neonates with prenatal substance exposure were also significantly younger (mean age, 28 years), were from disadvantaged socioeconomic quintiles, had higher rates of serious mental illness, and received less antenatal care than mothers of neonates without prenatal substance exposure (Table 1).

**Table 1.** Maternal and Perinatal Characteristics of Infants With and Without Exposure to Maternal Substance Use During Pregnancy, New South Wales, Australia, 2001-2020

Variable	No. (%)								
	Total children	Children not exposed to substance use during pregnancy^a^	Children exposed to substance use during pregnancy^b^	Maternal smoking during pregnancy	Maternal alcohol misuse/dependence^c^	Maternal smoking during pregnancy and maternal alcohol misuse/dependence^d^	Maternal drug misuse/dependence but no PDE or NAS diagnosis in the infant^e^	Children with PDE but no NAS^f^	Children with NAS^g^
No.	1 820 655	1 611 351	209 304	186 485	2781	1866	10 966	1260	5946
**Maternal characteristics**									
Maternal age, mean (SD), y	30.8 (5.5)	31.1 (5.3)	28.0 (6.1)	28.0 (6.1)	31.5 (5.8)	29.5 (6.4)	27.5 (6.2)	28.2 (6.2)	29.7 (5.9)
Gestation, mean (SD), wk	38.9 (1.9)	38.9 (1.9)	38.6 (2.2)	38.7 (2.2)	38.7 (2.3)	38.3 (2.5)	38.1 (2.7)	37.4 (2.8)	37.9 (2.4)
Young mother (<20 y)	52 425 (2.9)	33 894 (2.1)	18 531 (8.9)	16 748 (9.0)	85 (3.1)	131 (7.0)	1218 (11.1)	93 (7.4)	256 (4.3)
Self-identified Aboriginal and/or Torres Strait Islander	63 530 (3.5)	33 395 (2.1)	30 135 (14.4)	25856 (13.9)	148 (5.3)	400 (21.4)	2178 (19.9)	304 (24.1)	1249 (21.0)
SEIFA									
1st Quartile (most disadvantaged)	396 293 (21.8)	333 809 (20.7)	62 484 (29.9)	56 516 (30.3)	403 (14.5)	513 (27.5)	3037 (27.7)	318 (25.2)	1697 (28.5)
2nd Quartile	430 767 (23.7)	356 903 (22.1)	73 864 (35.3)	66 818 (35.8)	621 (22.3)	660 (35.4)	3594 (32.8)	414 (32.9)	1757 (29.6)
3rd Quartile	347 404 (19.1)	308 127 (19.1)	39 277 (18.8)	34 305 (18.4)	541 (19.5)	369 (19.8)	2524 (23.0)	260 (20.6)	1278 (21.5)
4th Quartile	286 021 (15.7)	265 308 (16.5)	20 713 (9.9)	18118 (9.7)	469 (16.9)	182 (9.8)	1012 (9.2)	176 (14.0)	756 (12.7)
5th Quartile (least disadvantaged)	339 066 (18.6)	328 476 (20.4)	10 590 (5.1)	8557 (8.5)	730 (26.3)	119 (6.4)	710 (6.5)	80 (6.4)	394 (6.6)
Late antenatal care (>20 wk at 1st visit)	153 673 (8.4)	120 356 (7.5)	33 317 (15.9)	28 594 (15.3)	238 (8.6)	361 (19.4)	2205 (20.1)	336 (26.7)	1583 (26.6)
Pregnancy complications									
Preeclampsia	23 431 (1.3)	21 331 (1.3)	2100 (1.00)	1845 (1.00)	32 (1.2)	14 (0.8)	120 (1.1)	12 (1.0)	77 (1.3)
Gestational diabetes	140 550 (7.7)	128 815 (8.0)	11 735 (5.6)	10741 (5.8)	150 (5.4)	100 (5.4)	477 (4.4)	40 (30.2)	227 (3.8)
Cesarean delivery	572 376 (31.4)	518 866 (32.2)	53 510 (25.6)	47 361 (25.4)	927 (33.3)	475 (25.5)	2717 (24.8)	346 (27.5)	1684 (28.3)
Serious maternal mental illness^h^	25 603 (1.4)	19 099 (1.2)	6504 (3.1)	3634 (2.0)	653 (2.0)	265 (14.2)	1496 (13.6)	69 (5.5)	387 (6.5)
Schizophrenia	1540 (0.1)	658 (0.0)	882 (0.4)	464 (0.3)	26 (0.9)	40 (2.1)	252 (2.3)	18 (1.4)	82 (1.4)
Schizophrenia/schizoaffective	2004 (0.1)	866 (0.1)	1138 (0.5)	566 (0.3)	32 (1.2)	53 (2.8)	355 (3.2)	23 (1.8)	109 (1.8)
Depression disorder	1676 (0.1)	1163 (0.1)	513 (0.3)	283 (0.2)	71 (2.6)	18 (1.0)	116 (1.1)	2 (0.2)	23 (0.4)
**Infant characteristics**									
Sex									
Male	935 807 (51.4)	828 155 (51.4)	107 652 (51.4)	95 931 (51.4)	1417 (50.1)	968 (51.9)	5494 (50.1)	666 (52.9)	3176 (53.4)
Female	884 848 (48.6)	783 196 (48.6)	101 652 (48.6)	90 554 (48.6)	1364 (49.1)	898 (48.1)	5472 (49.9)	594 (47.1)	2770 (46.6)
Gestational age									
<32 wk	24 630 (1.4)	19 736 (1.2)	4894 (2.3)	4029 (16.4)	65 (0.3)	60 (0.2)	454 (1.8)	89 (0.4)	197 (0.8)
<37 wk	126 262 (6.9)	104 314 (6.5)	21 948 (10.5)	17 941 (9.6)	238 (8.6)	263 (14.1)	1888 (17.2)	331 (26.3)	1287 (21.6)
Low birth weight (<2500 g)	107 777 (5.9)	83 990 (5.2)	23 787 (11.4)	19 384 (10.4)	215 (7.7)	308 (16.5)	2021 (18.4)	430 (34.1)	1429 (24.0)
Resuscitation required	494 610 (27.2)	428 519 (26.6)	66 091 (31.6)	57 979 (31.1)	1001 (36.0)	680 (36.4)	3627 (33.1)	517 (41.0)	2287 (38.5)
Apgar score <7 at 5 min	28 644 (1.6)	24 171 (1.5)	4473 (2.1)	3741 (2.0)	59 (2.1)	50 (2.7)	316 (2.9)	66 (5.2)	241 (4.1)
Admission to NICU	33 203 (1.8)	28 067 (1.7)	5136 (2.5)	4175 (2.2)	61 (2.2)	52 (2.8)	435 (4.0)	82 (6.5)	331 (5.6)
Death before discharge after birth	4501 (0.3)	3689 (0.2)	812 (0.4)	712 (0.4)	10 (0.4)	5 (0.3)	76 (0.7)	6 (0.5)	3 (0.1)
Out-of-home care									
Entry to out-of-home care	24 437 (1.3)	7056 (0.4)	17 381 (8.3)	11 494 (6.2)	146 (5.3)	405 (21.7)	2480 (22.6)	487 (38.7)	2369 (39.8)
Initial entry before age 6 mo	5668 (23.2)	1394 (19.8)	4274 (24.6)	2230 (19.4)	22 (15.1)	85 (21.0)	546 (22.0)	236 (48.5)	1155 (48.8)
Initial entry before age 1 y	7206 (29.5)	1818 (25.8)	5388 (31.0)	2968 (25.8)	30 (20.6)	116 (28.6)	710 (28.6)	263 (54.0)	1301 (54.9)
Type of out-of-home care									
Foster care^i^	9035 (37.0)	2731 (38.7)	6304 (36.3)	4090 (35.6)	53 (36.3)	143 (35.3)	849 (34.2)	201 (41.3)	968 (40.9)
Relative/kinship care^i^	13 555 (55.5)	3782 (53.6)	9773 (56.2)	6516 (56.7)	84 (57.5)	241 (59.5)	1417 (57.1)	257 (52.8)	1258 (53.1)
Other (eg, residential care)^i^	1847 (7.6)	543 (7.7)	1304 (7.5)	888 (7.7)	9 (6.2)	21 (5.2)	214 (8.6)	29 (6.0)	143 (6.0)

Abbreviations: NAS, neonatal abstinence syndrome; NICU, neonatal intensive care unit; PDE, prenatal drug exposure; *ICD-10-AM*, *International Classification of Diseases, Tenth Revision, Australian Modification*; SEIFA, Index of Relative Socio-economic Advantage and Disadvantage.

^a^ Infants with no known exposure to maternal smoking during pregnancy, maternal alcohol misuse/dependence, maternal drug misuse/dependence, and no PDE diagnosis (*ICD-10-AM* P04.4) or NAS diagnosis (*ICD-10-AM* P96.1).

^b^ Infants with a NAS diagnosis (*ICD-10-AM* P96.1); a PDE diagnosis (*ICD-10-AM* P04.4, newborn affected by maternal drug of addiction but no NAS); or exposed to maternal substance use, including alcohol, nicotine, opioids, cannabis, stimulants, sedatives, and/or hallucinogens based on *ICD-10-AM* F10-F19 (mental or behavioral disorders due to psychoactive substance abuse) recorded in the mother’s last hospital admission or an outpatient mental health care episode within 2 years before the birth.

^c^ Infants with exposure to maternal alcohol misuse/dependence based on *ICD-10-AM* F10-F19 (mental or behavioral disorders due to psychoactive substance abuse) recorded in the mother’s last hospital admission or an outpatient mental health care episode within 2 years before the birth.

^d^ Infants with exposure to maternal smoking during pregnancy and alcohol misuse/dependence based on *ICD-10-AM* F17.2 and F10.1-10.2, F10.9, or P04.3 (mental or behavioral disorders due to psychoactive substance abuse) recorded in the mother’s last hospital admission or an outpatient mental health care episode within 2 years before the birth.

^e^ Infants with exposure to maternal drug misuse/dependence—alcohol, nicotine, opioids, cannabis, stimulants, sedatives, and hallucinogens—based on *ICD-10-AM* F10-F19 (mental or behavioral disorders due to psychoactive substance abuse) recorded in the mother’s last hospital admission or an outpatient mental health care episode within 2 years before the birth but no PDE diagnosis or NAS diagnosis identified.

^f^ Infants with a PDE diagnosis but no NAS identified using *ICD-10-AM* P04.4.

^g^ Infants with a NAS diagnosis identified using *ICD-10-AM* P96.1.

^h^ Based on *ICD-10-AM* F10-F19 (mental or behavioral disorders due to psychoactive substance abuse) recorded in the mother’s last hospital admission or an outpatient mental health care within the 2 years prior to birth. Does not include diagnoses made by private mental health clinicians or those treated only by general practitioners.

^i^ Among children in out-of-home care placement.

### Characteristics of Out-of-Home Care Placement

The risk of out-of-home care involvement was higher for children with exposure to substance use during pregnancy than those without. Among those in out-of-home care, 1 in 4 children with prenatal substance exposure entered care by 6 months of age (4274 [24.6%]) compared to 1394 children without exposure (19.8%) (Table 1).

### Characteristics of Rehospitalizations

After birth discharge, 2 in 5 children with exposure to substance use during pregnancy had at least 1 inpatient readmission (90 433/209 304 [43.4%]) compared with 616 425/1 611 351 children (38.3%) without exposure (adjusted RR, 1.06; 95% CI, 1.06-1.07). This risk persisted up to age 20 years after adjusting for covariates (RR ranging from 1.05 to 1.12) (Table 2).

**Table 2.** Risk of Readmission for up to Age 20 Years by Types of Substance Use During Pregnancy

	Children not exposed to substance use during pregnancy; readmission, No. (%)^a^	Children exposed to substance use during pregnancy (all groups)^b^		Adjusted relative risk (95% CI)^c^^,^^j^^-^^k^						
		Readmission, No. (%)	Adjusted relative risk^c^^,^^j^^-^^k^ (95% CI)		Maternal smoking during pregnancy	Maternal alcohol use^d^	Maternal smoking during pregnancy and maternal alcohol use^e^	Maternal drug misuse/dependence but no PDE or NAS in the infant^f^	Children with PDE (but no NAS)^g^	Children with NAS^h^
No.	1 611 351	209 304	NA		186 485	2781	1866	10 966	1260	5946
Time since birth admission, y^i^										
5	616 425 (38.3)	90 433 (43.4)	1.06 (1.06-1.07)		1.05 (1.05-1.07)	1.01 (0.96-1.07)	1.07 (1.00-1.14)	1.01 (0.98-1.05)	1.12 (1.04-1.22)	1.21 (1.17-1.26)
6-10	241 292 (15.2)	38 921 (18.9)	1.05 (1.04-1.07)		1.05 (1.03-1.06)	1.00 (0.93- 1.09)	1.08 (0.98-1.20)	1.08 (1.03- 1.12)	1.22 (1.08- 1.38)	1.19 (1.12-1.26)
11-15	113 514 (7.2)	21 084 (10.3)	1.07 (1.06-1.09)		1.06 (1.04-1.08)	1.14 (1.02-1.28)	1.16 (1.03-1.32)	1.09 (1.03-1.16)	1.25 (1.06- 1.48)	1.27 (1.18- 1.37)
16-20	58 114 (3.6)	12 625 (6.1)	1.12 (1.10-1.14)		1.10 (1.07-1.12)	1.12 (0.97-1.29)	1.11 (0.93-1.31)	1.31 (1.22-1.40)	1.27 (1.02-1.60)	1.41 (1.30-1.59)

Abbreviations: *ICD-10-AM*, *International Statistical Classification of Diseases and Health Related Problems, Tenth Revision, Australian Modification*; NA, not applicable; NAS, neonatal abstinence syndrome; PDE, prenatal drug exposure.

^a^ Children with no known exposure to maternal smoking during pregnancy, maternal alcohol misuse/dependence, maternal drug misuse/dependence, and no PDE diagnosis (*ICD-10-AM* P04.4) or NAS diagnosis (*ICD-10-AM* P96.1).

^b^ Children with NAS diagnosis (*ICD-10-AM* P96.1); PDE diagnosis (*ICD-10-AM* P04.4; newborn affected by maternal drug of addiction but no NAS) or exposed to maternal substance use, including alcohol, nicotine, opioids, cannabis, stimulants, sedatives, and/or hallucinogens based on *ICD-10-AM* F10-F19 (mental or behavioral disorders due to psychoactive substance abuse) recorded in the mother’s last hospital admission or an outpatient mental health care episode within 2 years before the birth.

^c^ Risk compared with children not exposed to substance use during pregnancy.

^d^ Alcohol use here refers to misuse/dependence. This group includes children with exposure to maternal alcohol misuse/dependence based on *ICD-10-AM* F10-F19 (mental or behavioral disorders due to psychoactive substance abuse) recorded in the mother’s last hospital admission or an outpatient mental health care episode within 2 years before the birth.

^e^ Alcohol use here refers to misuse/dependence. This group includes children with exposure to maternal smoking during pregnancy and alcohol misuse/dependence based on *ICD-10-AM* F17.2 and F10.1-10.2, F10.9, or P04.3 (mental or behavioral disorders due to psychoactive substance abuse) recorded in the mother’s last hospital admission or an outpatient mental health care episode within 2 years before the birth.

^f^ Children with exposure to maternal drug misuse/dependence—alcohol, nicotine, opioids, cannabis, stimulants, sedatives, and hallucinogens—based on *ICD-10-AM* F10-F19 (mental or behavioral disorders due to psychoactive substance abuse) recorded in the mother’s last hospital admission or an outpatient mental health care episode within 2 years before the birth but no PDE or NAS diagnosis identified.

^g^ Children with PDE diagnosis but no NAS identified using *ICD-10-AM* P04.4.

^h^ Children with NAS identified using *ICD-10-AM* P96.1.

^i^ For children readmitted after birth discharge, the denominator is children alive at the start of each period.

^j^ Hospital episodes that resulted in a transfer were combined with previous episodes of care to calculate the risk of readmission with censoring for deaths. The values in the column are not true differences because of rounding.

^k^ Adjusted for young mother (<20 y), mothers who identified as Aboriginal and/or Torres Strait Islander, index of Relative Socio-economic Advantage and Disadvantage, diagnosis of serious mental health disorder based on last hospital admission or episode of mental health care in an ambulatory care setting prior to birth, infant sex, and year of study.

Readmission patterns varied by type of substance exposure. Children exposed to maternal smoking only during pregnancy, children with a prenatal substance exposure diagnosis, and those with NAS were more likely to be readmitted in all subsequent years compared with children who were not exposed to prenatal substance use. Children with NAS had the highest risk of readmission (RR, 1.21; 95% CI, 1.17-1.26) in the first 5 years, which persisted until early adulthood (RR range, 1.19-1.41) (Table 2). Mean LOS differed only slightly between children with exposure to substance use during pregnancy and those without exposure (approximately 1 day) (Table 3).

**Table 3.** Inpatient Hospital Length of Stay (LOS) and Cost for up to 20 Years of Age

	Children not exposed to substance use during pregnancy^a^		Children exposed to substance use during pregnancy (all groups 1-6)^b^			Children with NAS (group 6)^c^		
	Alive children, No.^d^	LOS, mean (SD), d^e^	Alive children^d^	Mean LOS difference (95% CI), d^f^	Adjusted excess cost (95% CI), A$^f^^-^^k^	Alive children^d^	Mean LOS difference (95% CI), d^f^	Adjusted excess cost (95% CI), A$^f^^-^^k^
Birth admission	1 611 351	4.2 (7.8)	209 304	0.50 (0.45-0.53)	1585 (1585-1586)	5946	10.91 (10.71-11.11)	19 404 (19 400-19 408)
Time since birth admission, y								
5	1 607 662	4.2 (13.8)	208 492	0.78 (0.67-0.87)	683 (533-833)	5943	1.72 (1.22-2.21)	2112 (1100-3124)
6-10	1 587 557	2.9 (11.1)	205 618	0.35 (0.23-0.47)	126 (77-174)	5894	0.93 (0.31-1.54)	454 (137-770)
11-15	1 587 264	4.1 (16.4)	205 539	0.63 (0.39-0.87)	165 (122-208)	5889	1.93 (0.71-3.15)	480 (197-763)
16-20	1 587 063	5.6 (21.5)	205 495	0.95 (0.54-1.36)	350 (304-397)	5884	1.68 (0.29-3.67)	694 (357-1030)

Abbreviations: AIHW, Australian Institute of Health and Welfare; AR-DRG, Australian Refined Diagnosis Related Groups; *ICD-10-AM*, *International Statistical Classification of Diseases and Health Related Problems, Tenth Revision, Australian Modification*; NAS, neonatal abstinence syndrome; PDE, prenatal drug exposure.

^a^ Children with no known prenatal exposure to maternal smoking, alcohol misuse/dependence, drug misuse/dependence, and no PDE diagnosis (*ICD-10-AM* P04.4) or NAS diagnosis (*ICD-10-AM* P96.1).

^b^ Children with NAS based on *ICD-10-AM* P96.1; PDE based on *ICD-10-AM* P04.4 (newborn affected by maternal drug of addiction but no NAS); or exposed to maternal substance use, including alcohol, nicotine, opioids, cannabis, stimulants, sedatives, and hallucinogens) based on *ICD-10-AM* F10-F19 (mental or behavioral disorders due to psychoactive substance abuse) recorded in the mother’s last hospital admission or an outpatient mental health care episode within 2 years before the birth.

^c^ Children with NAS identified using *ICD-10-AM* P96.1

^d^ Alive children are the number of children at the beginning of the period of interest.

^e^ LOS refers to the total LOS of each admission, including transfers. For LOS, the denominator is children with at least 1 admission during the period of interest. If a child was admitted and discharged on the same day, a LOS of 1 was assigned.

^f^ Mean LOS and excess hospital cost were compared with children not exposed to substance use during pregnancy.

^g^ US$1 = A$1.51.

^h^ Hospitalization costs were assigned to each episode using the AR-DRGs–specific mean national public hospital costs reported in the National Hospital Cost Data Collection Round associated with the year in which the episode of care occurred (ranging from AR-DRGs versions 6x-8.0).

^i^ Excess cost estimate using 2-part model adjusted for young mother (<20 y), mothers who identified as Aboriginal and/or Torres Strait Islander, index of Relative Socio-economic Advantage and Disadvantage, diagnosis of serious mental health disorder based on last hospital admission or episode of mental health care in an ambulatory care setting prior to birth, infant sex, and year of study.

^j^ In the 2-part model, the first part was a multivariate logistic regression model predicting the probability of inpatient readmission. The second part model the distribution of costs conditioned on having incurred positive costs (for each period of interest) using a generalized linear model with a γ distribution and a log link. The modified Park test was used to select the distribution and variance function for the generalized linear model.

^k^ All amounts were adjusted to 2019-2020 A$ using the AIHW inflation index.

### NAS vs Prenatal Drug Exposure Only

Compared with children with a prenatal drug exposure diagnosis, infants with NAS had longer hospital stay during birth admission (children with NAS, approximately 11 days vs 4.2 days for children with a prenatal drug exposure diagnosis; 95% CI, 2.40-4.21). After birth discharge, there were no differences in hospital use in all subsequent years between the 2 groups (eTable 2 in Supplement 1).

### Timing of and Reasons for Rehospitalization

Respiratory conditions (RR, 1.11; 95% CI, 1.09–1.12) and infections (RR, 1.12; 95% CI, 1.11–1.14) were the most common reasons for readmission among children exposed to prenatal substance use compared with those without exposure after adjusting for potential confounders (eFigure 3 in Supplement 1). Our study found that mental health/behavioral conditions requiring hospitalization were common among children with exposure to substance use during pregnancy (RR, 1.36; 95% CI, 1.33-1.41). In particular, mental health rehospitalization was 2-fold higher in children with NAS than those without exposure (RR, 2.28; 95% CI, 2.06-2.53). Notably, by age 1 year, children who had been exposed to substances (with or without NAS) were more likely to be readmitted for maltreatment, neglect, and misuse (eFigure 4 in Supplement 1).

### Costs of Birth and Rehospitalization

Longer hospital stays, greater need for special care or intensive care, and higher odds of readmission for children exposed to substance use during pregnancy were reflected in the higher hospital costs (adjusted mean difference, A$1585 per child; 95% CI, 1585-1586). Although children who were exposed to prenatal substance use incurred higher costs than those who were not, the cost burden was disproportionately higher for those with NAS. For example, at birth admission, children with NAS incurred A$19 404 (95% CI, 19 400-19 408) higher hospital costs compared with children who were not exposed to prenatal substance use. Estimates from the 2-part model showed that substance use during pregnancy remained associated with excess cost through young adulthood after adjusting for covariates (A$4176 per child; 95% CI, 3906-4445) (Figure). When extrapolated to the total registered births in Australia (approximately 300 000 births each year) using the incidence rate of children with prenatal substance exposure and the cost estimates from the 2-part model (Table 3), the total excess hospital costs attributed to substance use during pregnancy were estimated to be A$129.0 million in 2019 to 2020.

**Figure.**
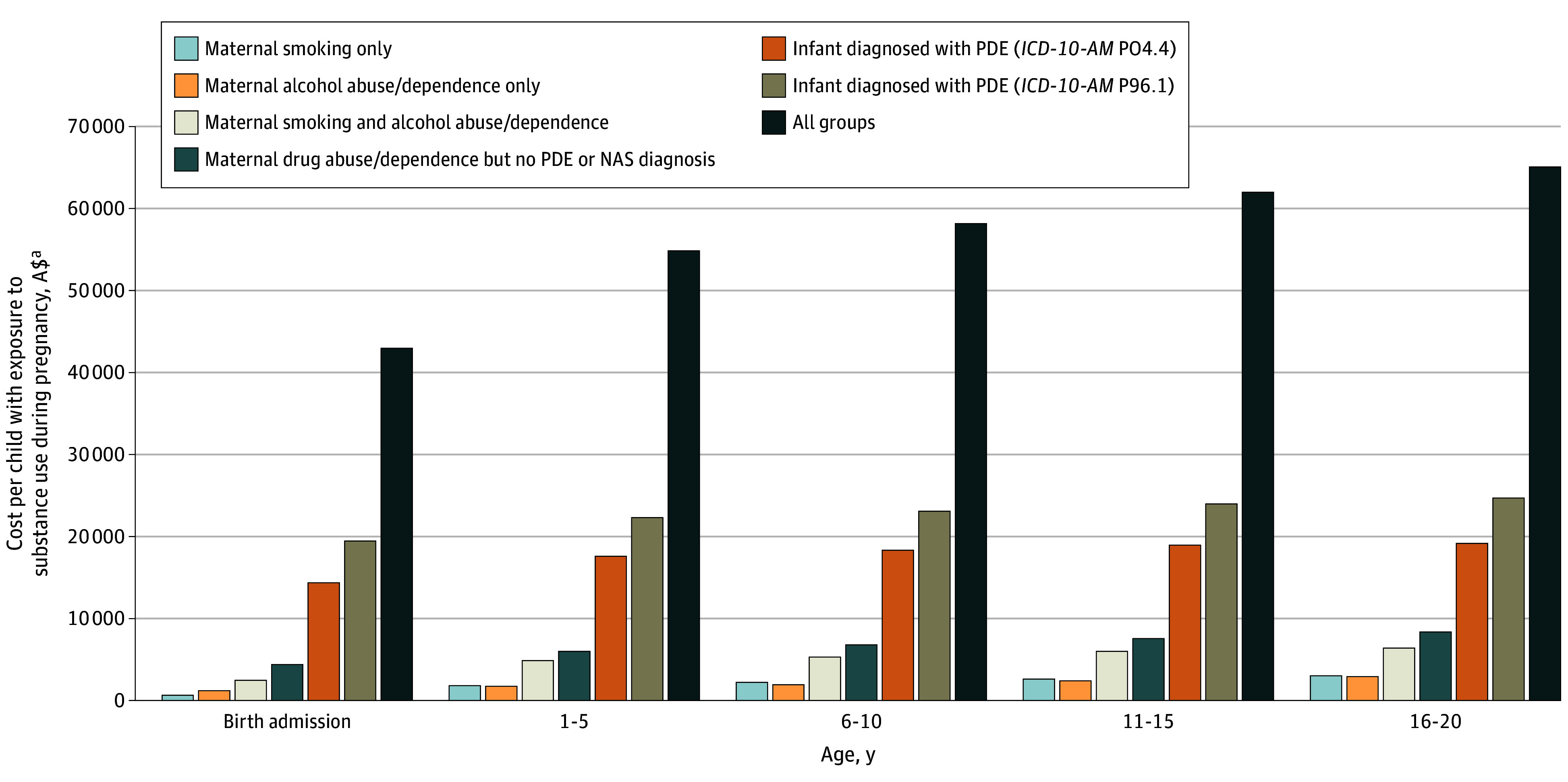
Cumulative Adjusted Excess Hospital Costs Per Child by Types of Substance Use During Pregnancy (SUP) From Birth Admission up to Age 20 Years Adjusted cost estimate using a 2-part model adjusted for young mother (aged <20 y), mothers who identified as Aboriginal and/or Torres Strait Islander, index of Relative Socio-economic Advantage and Disadvantage, diagnosis of serious mental health disorder based on last hospital admission or episode of mental health care in an ambulatory care setting prior to birth, infant sex, and year of study. *ICD-10-AM*, *International Classification of Diseases, Tenth Revision, Australian Modification*; NAS, neonatal abstinence syndrome; PDE, prenatal drug exposure ^a^US$1 = A$1.51.

### Causal Mediation via Out-of-Home Care

In the adjusted models, the natural direct effect showed that children with substance use during pregnancy were at higher risk of readmission, but any out-of-home care contact mediated the association of substance use during pregnancy and the readmission risk. For example, in the first 2 years of age, children with NAS were significantly more likely to be readmitted than children with no exposure (natural direct effect: adjusted RR, 1.28; 95% CI, 1.19-1.35) but any care arrangement mediated the risk of readmission by 27% (natural indirect effect: adjusted RR, 1.01; 95% CI, 0.98-1.02) (Table 4).

**Table 4.** Mediating Effect of Out-of-Home Care by Types of Substance Use During Pregnancy and Risk of Hospital Readmission and Costs

	Adjusted relative risk (95% CI)						Cumulative readmission cost (all groups), A$ million (95% CI)^f^
	Maternal smoking during pregnancy	Maternal alcohol misuse/dependence^a^	Maternal smoking during pregnancy and maternal alcohol misuse/ dependence^b^	Maternal drug misuse/dependence but no PDE or NAS diagnosis in the infant^c^	Children with PDE but no NAS^d^	Children with NAS^e^	
No.	186 485	2781	1866	10 966	1260	5946	
**Adjusted risk of readmission to 2 y of age^g^**							
Total effect^h^	1.15 (1.13-1.17)	0.98 (0.90-1.06)	1.23 (1.12-1.35)	1.02 (0.98-1.08)	1.21 (1.08-1.36)	1.27 (1.21-1.33)	67.5 (57.2-79.3)
Effect of out-of-home care (natural indirect effect)^i^	1.01 (1.01-1.02)	1.00 (0.99-1.03)	1.03 (1.00-1.07)	1.03 (1.02-1.05)	1.02 (0.97-1.07)	1.01 (0.98-1.02)	5.1 (4.6-10.4)
Effect of no out-of-home care (natural direct effect)^j^	1.13 (1.12-1.15)	0.96 (0.89-1.04)	1.22 (1.06-1.31)	1.03 (0.94-1.03)	1.27 (1.02-1.37)	1.28 (1.19-1.35)	59.8 (52.4-69.9)
**Adjusted risk of readmission to 5 y of age** ^g^							
Total effect^h^	1.10 (1.08-1.12)	0.99 (0.92-1.06)	1.08 (0.99-1.18)	1.00 (0.96-1.05)	1.17 (1.04-1.32)	1.20 (1.13-1.25)	133.2 (108.3-161.6)
Effect of out-of-home care (natural indirect effect)^i^	1.01 (1.01-1.02)	1.00 (0.99-1.01)	1.04 (1.01-1.08)	1.05 (1.03-1.07)	1.00 (0.96-1.05)	1.02 (1.00-1.05)	13.6 (11.7-26.7)
Effect of no out-of-home care (natural direct effect)^j^	1.08 (1.07-1.10)	0.98 (0.91-1.05)	1.03 (0.94-1.14)	0.95 (0.91-1.00)	1.16 (1.00-1.35)	1.16 (1.10-1.23)	112.2 (96.7-137.7)
**Adjusted risk of readmission to 8 y of age** ^g^							
Total effect^h^	1.10 (1.08-1.11)	0.98 (0.91-1.04)	1.06 (0.98-1.15)	1.04 (0.98-1.09)	1.16 (1.04-1.29)	1.23 (1.16-1.32)	213.8 (169.9-255.3)
Effect of out-of-home care (natural indirect effect)^i^	1.01 (1.01-1.02)	1.00 (0.99-1.01)	1.06 (1.02-1.11)	1.07 (1.03-1.10)	1.02 (0.97-1.07)	1.05 (1.01-1.10)	25.4 (20.4-41.7)
Effect of no out-of-home care (natural direct effect)^j^	1.08 (1.06-1.10)	0.97 (0.91-1.04)	1.00 (0.91-1.10)	0.97 (0.92-1.02)	1.13 (0.98-1.31)	1.18 (1.10-1.26)	173.3 (137.4-219.5)

Abbreviations: *ICD-10-AM*, *International Statistical Classification of Diseases and Health Related Problems, Tenth Revision, Australian Modification*; NAS, neonatal abstinence syndrome; PDE, prenatal drug exposure.

^a^ Children with exposure to maternal alcohol misuse/dependence based on *ICD-10-AM* F10-F19 (mental or behavioral disorders due to psychoactive substance abuse) recorded in the mother’s last hospital admission or an outpatient mental health care episode within 2 years before the birth.

^b^ Children with exposure to maternal smoking during pregnancy and alcohol misuse/dependence based on *ICD-10-AM* F17.2 and F10.1-F10.2, F10.9, or P04.3 (mental or behavioral disorders due to psychoactive substance abuse) recorded in the mother’s last hospital admission or an outpatient mental health care episode within 2 years before the birth.

^c^ Children with exposure to maternal drug misuse/dependence—alcohol, nicotine, opioids, cannabis, stimulants, sedatives, and hallucinogens—based on *ICD-10-AM* F10-F19 (mental or behavioral disorders due to psychoactive substance abuse) recorded in the mother’s last hospital admission or an outpatient mental health care episode within 2 years before the birth but no PDE or NAS diagnosis identified in the infant.

^d^ Children with PDE but no NAS identified using *ICD-10-AM* P04.4.

^e^ Children with NAS identified using *ICD-10-AM* P96.1.

^f^ Cumulative hospital costs were based on excess risk of inpatient readmission by types of prenatal drug exposure and pathways with direct and indirect via out-of-home care as mediator. US$1 = A$1.51.

^g^ Relative risk and 95% CI adjusted for young mother (<20 y), mothers who identified as Aboriginal and/or Torres Strait Islander, index of Relative Socio-economic Advantage and Disadvantage, diagnosis of serious mental health disorder based on last hospital admission or episode of mental health care in an ambulatory care setting prior to birth, infant sex, and year of study.

^h^ Total effect indicates the effect of substance use during pregnancy on the risk of readmission as it stands, without taking into account any mediating effect of out-of-home care. The total effect of substance use during pregnancy (as an exposure) on risk of readmission (outcome) comprised the natural direct effect and the natural indirect effect. Total direct effect, natural direct effect, and natural indirect effect were calculated using the mediate command in Stata version 18 (StataCorp).

^i^ Natural indirect effect indicates the effect of substance use during pregnancy on the risk of readmission via a mediator (out-of-home care) if all children had received out-of-home care.

^j^ Natural direct effect indicates the effect of substance use during pregnancy on the risk of readmission if all children exposed to substance use during pregnancy had not received out-of-home care.

Similarly, based on excess relative risk of readmission in the mediation analyses and associated readmission costs, the cumulative hospital costs associated with substance use during pregnancy was estimated to be A$213.8 million (95% CI, 169.9 million-255.3 million). However, any out-of-home care contact mediated the readmission risk and associated cost to health care system of A$25.4 million (95% CI, 20.4 million-41.7 million) compared with A$173.3 million (95% CI, 137.4 million-219.5 million) in the absence of care engagement.

## Discussion

To our knowledge, this cohort study is the first study to examine hospital costs associated with birth, rehospitalization, and out-of-home care in children with exposure to substance use during pregnancy, including NAS. Our findings show that children with exposure to substance use during pregnancy, with or without NAS diagnosis, are at higher risk of adverse birth outcomes and hospital costs than children without exposure, even until age 20 years. The estimated total cost associated with substance use during pregnancy (A$129 million) was comparable to annual government spending for Australian children with neurological conditions (A$121.0 million), childhood cancer and other neoplasm (A$131.6 million), and musculoskeletal disorders (A$147.5 million) in 2019 to 2020.^28^

A notable finding from our study showed that any engagement with out-of-home care, an indicator of child protection surveillance, could almost completely mitigate readmission rates and costs for children exposed to substance use during pregnancy. We emphasize that this does not suggest that out-of-home care should be an intervention but rather that engagement in preventive and supportive services could have beneficial health effects, such as access to timely treatment for vulnerable children.^12^ This is important given the considerably higher cost burden associated with substance use during pregnancy, particularly among children with a history of NAS. Indeed, after the neonatal period, children with NAS remained at higher risk of inpatient readmission up to early adulthood. Investing in evidence-based services (eg, nurse-led home visiting programs that follow up with children at many time points over their early years), could prevent or mitigate the impact of exposure to substance use during pregnancy beyond early life.^29^

The causes of readmissions observed were myriad and could be due to many factors, including social and economic problems. However, previous studies have shown that low parental health literacy and socioeconomic status were associated with poor use and access to appropriate primary care services.^30,31,32^ Disadvantaged families are more likely to delay primary treatment and default to tertiary care services, as these services do not incur out-of-pocket fees in Australia.^32,33^ It is possible then that when children with exposure to substance use during pregnancy attend hospitals, they are sicker than those who have had their basic health needs met.

In particular, admissions for mental health/behavioral problems were higher in the group exposed to substance use during pregnancy. The cause for this is unknown, but we speculate that vulnerable children may have difficulties accessing early mental health support if their family life is not supportive.^33^ Delayed professional support is associated with worsened mental health status.^34^ Programs such as For Baby’s Sake in the UK,^35^ Mothers and Babies in the US,^36^ and the Substance Use in Pregnancy and Parenting Services in Australia^37^ have shown promising results in identifying and addressing the complex needs of this population. The importance of early identification, intervention, and prevention strategies cannot be overstated, especially for families with greater disadvantage, as this has enormous potential to disrupt the accumulation of adversity over the life course of generations.^38^

Consistent with earlier findings, the high prevalence of maltreatment, including parental neglect and abuse among children younger than 1 year, in our study is of great concern.^4^ Whether this is a direct result of direct substance use or a combination of social and environmental adversities that increased the risk of child harm is uncertain, but the question cannot be ignored. It is well established that experiences in early childhood can adversely affect health and developmental outcomes and may contribute to higher rates of poor mental health and risk of substance use in adulthood.^38^ In Australia, the National Framework for Protecting Australia’s Children 2009 to 2020 aims to protect vulnerable children and families,^39^ but ongoing monitoring is required to assess the effectiveness of such frameworks especially for children with exposure to substance use during pregnancy.^40^

### Strengths and Limitations

A key strength of this study is the use of population-level registry data for the analysis of trajectories of health care use and cost impact. The mediation framework provided a novel approach and showed that active engagement and support are associated with improved health outcomes for children with exposure to substance use during pregnancy.

Our study has limitations. The impact of social desirability bias (ie, underreporting of substance use during pregnancy) cannot be dismissed and, unfortunately, cannot be determined directly from administrative data. However, due to the nonpunitive nature of the Australian health and welfare system, diagnoses of children with exposure to substance use during pregnancy, especially in the most vulnerable families, are reliable and mirror street trends and other social factors accurately.^41^ Children were classified using *ICD-10-AM* F10-F19 codes relating to maternal admissions for substance misuse/dependence (licit and illicit drugs). It is possible that this identified only the most severe cases, wherein individuals are more likely to be sicker and require inpatient admission. Furthermore, even though our analyses adjusted for confounding variables, unmeasurable characteristics (eg, environmental factors [distances]) may influence access to hospital-based services and we did not have information on primary health services, such as outpatient clinic visits, or those who may have since moved interstate.

As our study only assessed the influence of out-of-home care on the risk of substance use during pregnancy on readmission, future research should evaluate other entrenched models of care for children exposed to substance use during pregnancy.^29^ Additionally, we were unable to determine from our administrative data if maternal drug use continued following birth, but it is well established that women who used substances prior to conception are likely to continue use during and after pregnancy.^42,43^

## Conclusion

In this study, children with exposure to substance use during pregnancy, particularly those with NAS, had increased health care needs and generated more health care–associated costs even into young adulthood. However, we also show that any contact with out-of-home care was associated with decreased costs. Further study needs to be done to elucidate the underlying mechanisms for this finding.

## Notes

Supplement 1. eTable 1. Summary of Databases in Record Linkage eTable 2. Inpatient hospital utilisation and costs for up to 20 years―children with PDE diagnosis (Group 5) and children with NAS (Group 6) eFigure 1. Patient flow chart eFigure 2. Directed Acyclic Graph eFigure 3. Adjusted Risk of Re-admission to early adulthood eFigure 4. Median time (days) to first readmission for specific conditions

Supplement 2. Data sharing statement

## References

[1] Ramphul K, Mejias SG, Joynauth J. An update on the burden of neonatal abstinence syndrome in the United States. Hosp Pediatr. 2020;10(2):181-184. doi:10.1542/hpeds.2019-0221 31932280

[2] Winkelman TNA, Villapiano N, Kozhimannil KB, Davis MM, Patrick SW. Incidence and costs of neonatal abstinence syndrome among infants with Medicaid: 2004–2014. Pediatrics. 2018;141(4):e20173520. doi:10.1542/peds.2017-3520 29572288 PMC5869343

[3] Rees P, Stilwell PA, Bolton C, . Childhood health and educational outcomes after neonatal abstinence syndrome: a systematic review and meta-analysis. J Pediatr. 2020;226:149-156.e16. doi:10.1016/j.jpeds.2020.07.013 32659230

[4] Uebel H, Wright IM, Burns L, . Reasons for rehospitalization in children who had neonatal abstinence syndrome. Pediatrics. 2015;136(4):e811-e820. doi:10.1542/peds.2014-2767 26371197

[5] Liu G, Kong L, Leslie DL, Corr TE. A longitudinal healthcare use profile of children with a history of neonatal abstinence syndrome. J Pediatr. 2019;204:111-117.e1. doi:10.1016/j.jpeds.2018.08.032 30270164

[6] Corr TE, Xing X, Liu G. Longitudinal health care utilization of Medicaid-insured children with a history of neonatal abstinence syndrome. J Pediatr. 2021;233:82-89.e1. doi:10.1016/j.jpeds.2021.01.067 33545189

[7] Malthaner LQ, Jetelina KK, Loria H, McLeigh JD. Healthcare utilization among children with a history of neonatal opioid withdrawal syndrome: a matched cohort study. Child Abuse Negl. 2022;134:105934. doi:10.1016/j.chiabu.2022.105934 36302288

[8] Leyenaar JK, Schaefer AP, Wasserman JR, Moen EL, O’Malley AJ, Goodman DC. Infant mortality associated with prenatal opioid exposure. JAMA Pediatr. 2021;175(7):706-714. doi:10.1001/jamapediatrics.2020.6364 33843963 PMC8042571

[9] Stephansson O, Kieler H, Haglund B, . Selective serotonin reuptake inhibitors during pregnancy and risk of stillbirth and infant mortality. JAMA. 2013;309(1):48-54. doi:10.1001/jama.2012.153812 23280224

[10] Kieviet N, Dolman KM, Honig A. The use of psychotropic medication during pregnancy: how about the newborn? Neuropsychiatr Dis Treat. 2013;9:1257-1266. doi:10.2147/NDT.S36394 24039427 PMC3770341

[11] Taplin S, Mattick RP. Mothers in methadone treatment and their involvement with the child protection system: a replication and extension study. Child Abuse Negl. 2013;37(8):500-510. doi:10.1016/j.chiabu.2013.01.003 23428166

[12] Gnanamanickam ES, Brown DS, Armfield JM, Segal L. Excess hospital costs incurred by individuals with child abuse and neglect history in South Australia: a birth-cohort study. Prev Med. 2023;166:107378. doi:10.1016/j.ypmed.2022.107378 36493867

[13] Dubois-Comtois K, Bussières EL, Cyr C, . Are children and adolescents in foster care at greater risk of mental health problems than their counterparts? a meta-analysis. Child Youth Serv Rev. 2021;127:106100. doi:10.1016/j.childyouth.2021.106100

[14] Turney K, Wildeman C. Mental and physical health of children in foster care. Pediatrics. 2016;138(5):e20161118. doi:10.1542/peds.2016-1118 27940775

[15] Rebbe R, Nurius PS, Courtney ME, Ahrens KR. Adverse childhood experiences and young adult health outcomes among youth aging out of foster care. Acad Pediatr. 2018;18(5):502-509. doi:10.1016/j.acap.2018.04.011 29709622 PMC6035089

[16] Arora N, Kaltner M, Williams J. Health needs of regional Australian children in out-of-home care. J Paediatr Child Health. 2014;50(10):782-786. doi:10.1111/jpc.12637 25288239

[17] Irvine K, Hall R, Taylor L. A profile of the Centre for Health Record Linkage. Int J Popul Data Sci. 2019;4(2):1142. 34095543 10.23889/ijpds.v4i1.1142

[18] Independent Hospital Pricing Authority. National hospital cost data collection report 2012-13. round 17. 2015. Accessed June 18, 2024. https://www.ihacpa.gov.au/resources/national-hospital-cost-data-collection-nhcdc-public-sector-report-2012-13

[19] Independent Hospital Pricing Authority. National hospital cost data collection Australian public hospitals cost report 2013-14, round 18. 2016. Accessed June 18, 2024. https://www.ihacpa.gov.au/resources/national-hospital-cost-data-collection-nhcdc-public-sector-report-2013-14

[20] Independent Hospital Pricing Authority. National hospital cost data collection report 2016-17 round 21. 2019. Accessed June 18, 2024. https://www.ihacpa.gov.au/resources/national-hospital-cost-data-collection-private-hospital-report-2016-17

[21] Australian Bureau of Statistics. Australian statistical geography standard (ASGS): volume 5—remoteness structure. 2011. Accessed June 18, 2024. https://www.abs.gov.au/statistics/statistical-geography/australian-statistical-geography-standard-asgs

[22] Azuine RE, Ji Y, Chang HY, . Prenatal risk factors and perinatal and postnatal outcomes associated with maternal opioid exposure in an urban, low-income, multiethnic US population. JAMA Netw Open. 2019;2(6):e196405. doi:10.1001/jamanetworkopen.2019.6405 31251378 PMC6604084

[23] O’Donnell M, Nassar N, Leonard H, . Increasing prevalence of neonatal withdrawal syndrome: population study of maternal factors and child protection involvement. Pediatrics. 2009;123(4):e614-e621. doi:10.1542/peds.2008-2888 19336352

[24] Mihaylova B, Briggs A, O’Hagan A, Thompson SG. Review of statistical methods for analysing healthcare resources and costs. Health Econ. 2011;20(8):897-916. doi:10.1002/hec.1653 20799344 PMC3470917

[25] VanderWeele T. Explanation in Causal Inference: Methods for Mediation and Interaction. Oxford University Press; 2015.

[26] Cole SR, Platt RW, Schisterman EF, . Illustrating bias due to conditioning on a collider. Int J Epidemiol. 2010;39(2):417-420. doi:10.1093/ije/dyp334 19926667 PMC2846442

[27] Baron RM, Kenny DA. The moderator-mediator variable distinction in social psychological research: conceptual, strategic, and statistical considerations. J Pers Soc Psychol. 1986;51(6):1173-1182. doi:10.1037/0022-3514.51.6.1173 3806354

[28] Australian Institute of Health and Welfare (AIHW) Australian Government. Disease expenditure Australia 2019-20. 2021. Accessed June 18, 2024. https://www.aihw.gov.au/reports/health-welfare-expenditure/disease-expenditure-in-australia-2019-20/contents/summary

[29] Oei JL, Azim SI, Lee E, . Substance use during pregnancy, birth and the postnatal period. SaxInstitute. 2021. Accessed June 18, 2024. https://www.saxinstitute.org.au/wp-content/uploads/21.06_Evidence-Check_Substance-use-during-pregnancy-birth-and-the-postnatal-period.pdf

[30] Zaidman EA, Scott KM, Hahn D, Bennett P, Caldwell PH. Impact of parental health literacy on the health outcomes of children with chronic disease globally: a systematic review. J Paediatr Child Health. 2023;59(1):12-31. doi:10.1111/jpc.16297 36536542

[31] Bull C, Howie P, Callander EJ. Inequities in vulnerable children’s access to health services in Australia. BMJ Glob Health. 2022;7(3):e007961. doi:10.1136/bmjgh-2021-007961 35346955 PMC8961130

[32] Eapen V, Walter A, Guan J, ; The ‘Watch Me Grow’ Study Group. Maternal help-seeking for child developmental concerns: associations with socio-demographic factors. J Paediatr Child Health. 2017;53(10):963-969. doi:10.1111/jpc.13607 28661061

[33] Sawyer MG, Reece CE, Sawyer AC, Johnson SE, Hiscock H, Lawrence D. Access to health professionals by children and adolescents with mental disorders: are we meeting their needs? Aust N Z J Psychiatry. 2018;52(10):972-982. doi:10.1177/0004867418760713 29498290

[34] Griffen A, McIntyre L, Belsito JZ, . Perinatal mental health care in the United States: an overview of policies and programs. Health Aff. 2021;40(10):1543-1550. doi:10.1377/hlthaff.2021.00796 34606347

[35] Domoney J, Fulton E, Stanley N, . For baby’s sake: Intervention development and evaluation design of a whole-family perinatal intervention to break the cycle of domestic abuse. J Fam Violence. 2019;34:539-551. doi:10.1007/s10896-019-00037-3

[36] The Mothers & Babies Program. The Mothers & Babies Program website. Accessed December 31, 2023. https://www.mothersandbabiesprogram.org/

[37] Coupland H, Moensted ML, Reid S, . Developing a model of care for substance use in pregnancy and parenting services, Sydney, Australia: service provider perspectives. J Subst Abuse Treat. 2021;131:108420. doi:10.1016/j.jsat.2021.108420 34098295

[38] Hughes K, Bellis MA, Hardcastle KA, . The effect of multiple adverse childhood experiences on health: a systematic review and meta-analysis. Lancet Public Health. 2017;2(8):e356-e366. doi:10.1016/S2468-2667(17)30118-4 29253477

[39] Babington B. National framework for protecting Australia’s children: perspectives on progress and challenges. Fam Matters. 2011;89:11-20. Accessed June 18, 2024. https://aifs.gov.au/research/family-matters/no-89/national-framework-protecting-australias-children

[40] McKibbin G, Humphreys C. Future directions in child sexual abuse prevention: an Australian perspective. Child Abuse Negl. 2020;105:104422. doi:10.1016/j.chiabu.2020.104422 32122641

[41] Pong KM, Abdel-Latif ME, Lui K, . The temporal influence of a heroin shortage on pregnant drug users and their newborn infants in Sydney, Australia. Aust N Z J Obstet Gynaecol. 2010;50(3):230-236. doi:10.1111/j.1479-828X.2010.01146.x 20618239

[42] Young-Wolff KC, Sarovar V, Alexeeff SE, . Trends and correlates of self-reported alcohol and nicotine use among women before and during pregnancy, 2009-2017. Drug Alcohol Depend. 2020;214:108168. doi:10.1016/j.drugalcdep.2020.108168 32736316 PMC7423641

[43] Chang G, McNamara TK, Orav EJ, Wilkins-Haug L. Alcohol use by pregnant women: partners, knowledge, and other predictors. J Stud Alcohol. 2006;67(2):245-251. doi:10.15288/jsa.2006.67.245 16562406 PMC1540454

