# Supplementary material for: Health Care Needs and Costs for Children Exposed to Prenatal Substance Use to Adulthood
Source: JAMA Pediatr. 2024 Jul 22;178(9):888–98. doi: 10.1001/jamapediatrics.2024.2281 (PMC11264092; doi:10.1001/jamapediatrics.2024.2281)
Supplement: Supplement 1. — eTable 1. Summary of Databases in Record Linkage eTable 2. Inpatient hospital utilisation and costs for up to 20 years―children with PDE diagnosis (Group 5) and children with NAS (Group 6) eFigure 1. Patient flow chart eFigure 2. Directed Acyclic Graph eFigure 3. Adjusted Risk of Re-admission to early adulthood eFigure 4. Median time (days) to first readmission for specific conditions [file jamapediatr-e242281-s001.pdf]

Supplemental Online Content

Lee E, Schofield D, Dronavalli M, et al. Health care needs and costs for children exposed to prenatal substance use to adulthood. *JAMA Pediatr*. Published online July 22, 2024. doi:10.1001/jamapediatrics.2024.2281

eTable 1. Summary of Databases in Record Linkage

eTable 2. Inpatient hospital utilisation and costs for up to 20 years—children with PDE diagnosis (Group 5) and children with NAS (Group 6)

eFigure 1. Patient flow chart

eFigure 2. Directed Acyclic Graph

eFigure 3. Adjusted Risk of Re-admission to early adulthood

eFigure 4. Median time (days) to first readmission for specific conditions

This supplemental material has been provided by the authors to give readers additional information about their work.

© 2024 Lee E et al. *JAMA Pediatrics.*

eTable 1. Summary of Databases in Record Linkage

Supplementary Table 1. Summary of Databases in Record Linkage		
**Database**	**Information Available**	**Dates**
**PRIMARY DATABASES**		
**NSW Perinatal Data Collection (PDC Babies)**	▪ Maternal demographic information<br>▪ Maternal medical information<br>▪ Maternal obstetric information<br>▪ Delivery information<br>▪ Infant demographic information, including gestation, and birth weight, condition, APGAR score and resuscitation details	1 Jul 2001 to 31 Dec 2020
**NSW Perinatal Data Collection (PDC Mothers)**		1 Jul 2001 to 31 Dec 2020
**LINKED DATABASES**		
**NSW Admitted Patient Data Collection (APDC)**	▪ Information on separations (discharges, transfers and deaths) and health service facility use for NSW residents within and outside NSW<br>▪ Demographic details and administrative items<br>▪ Diagnoses (ICD-10-AM) – up to 50 diagnoses associated with each episode of care (included NAS [P96·1], newborn affected by maternal drugs of addiction [P04·4], and other maternal drug/alcohol related disorders [F10-F19]).	1 Jul 2001 to 31 Dec 2021
**NSW Mental Health Ambulatory Data Collection (MH AMB)**	▪ Demographic information<br>▪ Diagnosis (ICD-10-AM), used to identify maternal diagnoses relating to alcohol/drug abuse/dependence.	1 Jul 2001 to 31 Dec 2021
**NSW Registry for Births, Deaths & Marriages (RBDM)**	▪ Demographic information<br>▪ Date of birth and date of death (used to calculate age of death)	1 Jul 2001 to 31 Dec 2021
**NSW Cause of Death Unit Record File (COD URF)**	▪ Demographic information<br>▪ Cause of death (ICD-10-AM)	1 Jul 2001 to 31 Dec 2020
**NSW Family and Community Services Dataset – KiDS Data Collection (DCJ KiDS)**	▪ Demographic information placement date, duration of care, and type of care (e.g. Foster Care, Relative/Kinship Care, residential care)	18 Jul 2001 to 30 Jun 2021

The primary database is the Perinatal Data Collection (PDC), which provides information on maternal, and infant born between 1st July 2001 and 31st December 2020 in NSW. The PDC was linked to other population datasets including the NSW Admitted Patient Data Collection (APDC) database which provided information on all hospital separations (discharges, transfers, and deaths) in public hospital in NSW, Registry of Births, Deaths and Marriages (RBDM) & Cause of Death Unit Record File (COD URF – death details); NSW Mental Health Ambulatory Data Collection (MH AMB – maternal outpatient mental health diagnoses); and NSW Family and Community Services Dataset – KiDS Data Collection (OOHC information).

Abbreviation: NSW, New South Wales; PDC, Perinatal Data Collection; COD: Cause of Death; NSW, New South Wales; ICD-10-AM, International Statistical Classification of Diseases and Related Problems, Tenth Revision, Australian Modification

© 2024 Lee E et al. *JAMA Pediatrics.*

eTable 2: Inpatient hospital utilisation and costs for up to 20 years—children with a PDE diagnosis (G*roup 5)*[a] and children with NAS (group 6)[b]

	Children with NAS Group 6[a]				Children with PDE (*but no NAS diagnosis*) Group 5[b]				RR of re-admission (Group 6 versus 5)		Crude difference (Group 6 versus 5)	
	Children[c] N	Children re-admitted[d] n (%)	LOS[e], Mean (SD), day	Mean cost[f] A$ (SD)	Children[c] N	Children re-admitted[d] no. (%)	LOS[e], Mean (SD), days	Mean cost[f] A$ (SD)	Unadjusted RR (95% CI)	Adjusted RR[g] (95%CI)	Mean LOS[e], (95%CI), days	Mean cost[f] A$ (95%CI)
Birth admission	5,946	-	**15.0** (14.6)	**26,319** (31,886)	1,260	-	**11.7** (16.1)	**21,023** (40,025)	-	-	**3.3** (2.40,4.21)	**5,296** (3,261, 7,331)
After birth discharge–5th y	5,943	**2,974** (50.0)	**5.9** (10.9)	**6,230** (20,467)	1,254	**573** (45.7)	**6.6** (13.0)	**6,609** (21,685)	**1.09** (1.00-1.20)	**1.06** (0.97, 1.17)	**-0.6** (-1.70, 0.3)	**-378** (-1638, 881)
6th -10th y	5,894	**1,231** (20.9)	**3.9** (10.6)	**1,699** (9,653)	1,245	**262** (21.0)	**3.3** (7.3)	**1,648** (9,570)	**1.00** (0.86- 1.13)	**0.97** (0.85, 1.11)	**0.6** (-0.80, 1.9)	**48** (-540, 639)
11th- 15th y	5,889	**699** (11.9)	**6.0** (20.1)	**1,472** (14,511)	1,244	**139** (11.2)	**4.8** (10.0)	**1,194** (8,236)	**1.06** (0.88-1.27)	**1.01** (0.84, 1.22)	**1.2** (-2.2, 4.6)	**277** (-556, 1,111)
16th- 20thy	5,884	**452** (7.7)	**7.3** (17.6)	**1,018** (8,180)	1,243	**82** (6.6)	**4.2** (7.2)	**553** (4,172)	**1.16** (0.92-1.47)	**1.15** (0.90, 1.46)	**3.1** (-0.77, 7.0)	**464** (-2.7, 931)

Notes: [a] Newborn diagnosed with NAS (ICD-10-AM code P96.1)
[b] Newborn with clinically observed manifestations of PDE but no NAS (ICD-10-AM code P04.4).
[c] Number of children alive at the start of each period.
[d] For children re-admitted after birth discharge, the denominator is children alive at the start of each period.
[e] Length of stay (LOS) refers to the total LOS of each admission including transfers. For LOS, the denominator is children with at least one admission during the period of interest. If a child was admitted and discharged on the same day, a LOS of 1 was assigned.
[f] Mean cost is per alive child at the beginning of the period.
[g] Adjusted for young mother (aged < 20), self-identified Aboriginal and Torres Strait Islander heritage, index of Relative Socio-economic Advantage and Disadvantage, diagnosis of serious mental health disorder based on last hospital admission or episode of mental health care in an ambulatory care setting prior to birth, infant's gender and year of study. The values in the column are not true differences because of rounding.

Abbreviations: PDE: Prenatal drug exposure, NAS: Neonatal Abstinence Syndrome; RR: relative risk, LOS, length of stay; SD: Standard Deviation; ICD-10-AM: International Statistical Classification of Diseases and Related Problems, Tenth Revision, Australian Modification; A$: Australian dollars; 95%CI: 95% confidence interval with two tailed comparisons.

© 2024 Lee E et al. *JAMA Pediatrics.*

eFigure 1. Patient Flow Chart

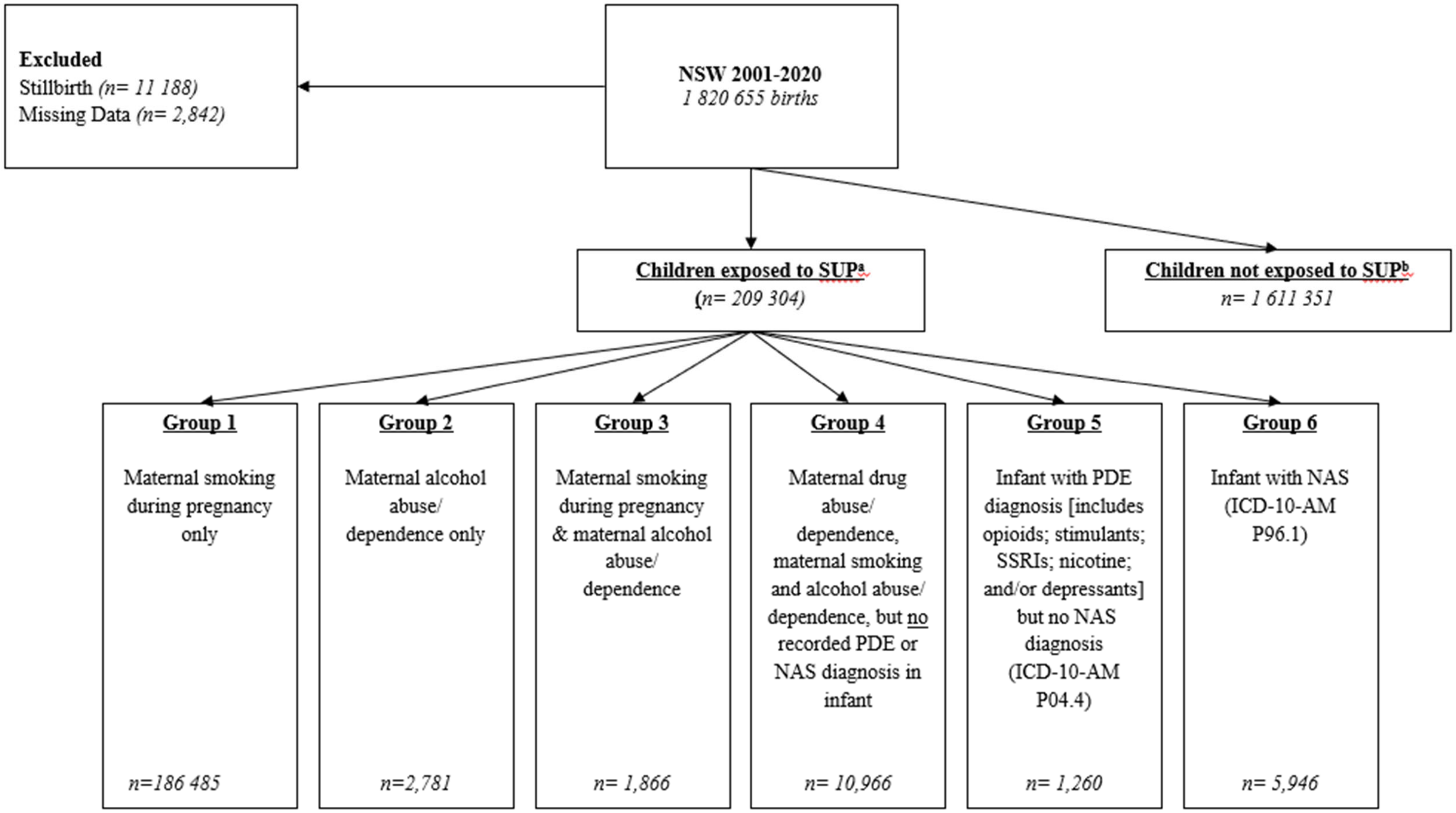

[a] Children exposed to SUP = newborns with NAS diagnosis (ICD-10-AM P96.1), PDE diagnosis (ICD-10-AM P04.4, *newborn affected by maternal drug of addiction but no NAS*) or exposed to maternal substance use including alcohol, nicotine, opioids, cannabis, stimulants, sedatives and/or hallucinogens based on ICD-10-AM F10-F19 (*mental or behavioural disorders due to psychoactive substance abuse*) recorded in the mother's last hospital admission or an outpatient mental health care episode within two years before birth.

[b] Children not exposed to SUP= Children with no known exposure to maternal smoking during pregnancy, maternal alcohol abuse/dependence, maternal drug abuse/dependence, no PDE diagnosis (ICD-10-AM P04.4) or NAS diagnosis (ICD-10-AM P96.1)

Abbreviations: SUP, substance use during pregnancy; PDE, prenatal drug exposure; NAS, Neonatal Abstinence Syndrome; NSW, New South Wales; ICD-10-AM, International Statistical Classification of Disease and Related Problems (10th Edition) Australian Modification.

© 2024 Lee E et al. *JAMA Pediatrics.*

eFigure 2: Directed Acyclic Graph

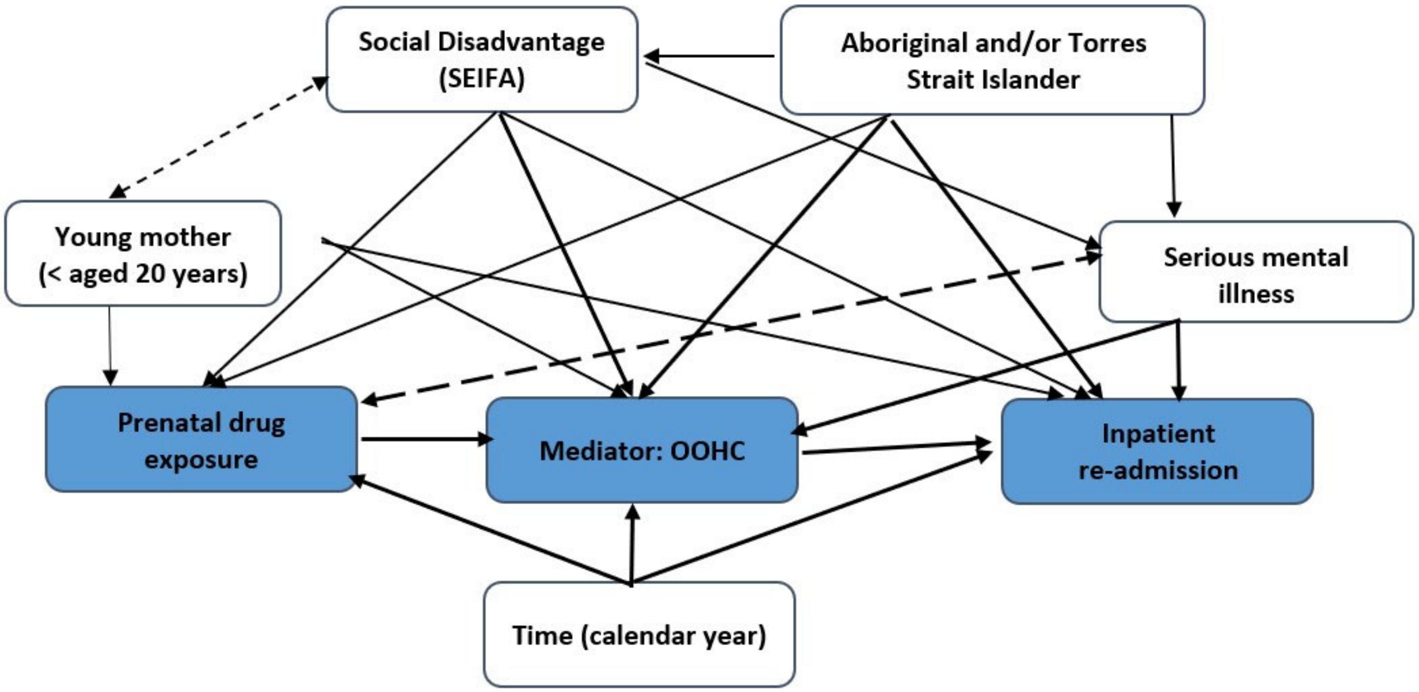

© 2024 Lee E et al. *JAMA Pediatrics.*

eFigure 3: Adjusted Risk of Readmission to early adulthood

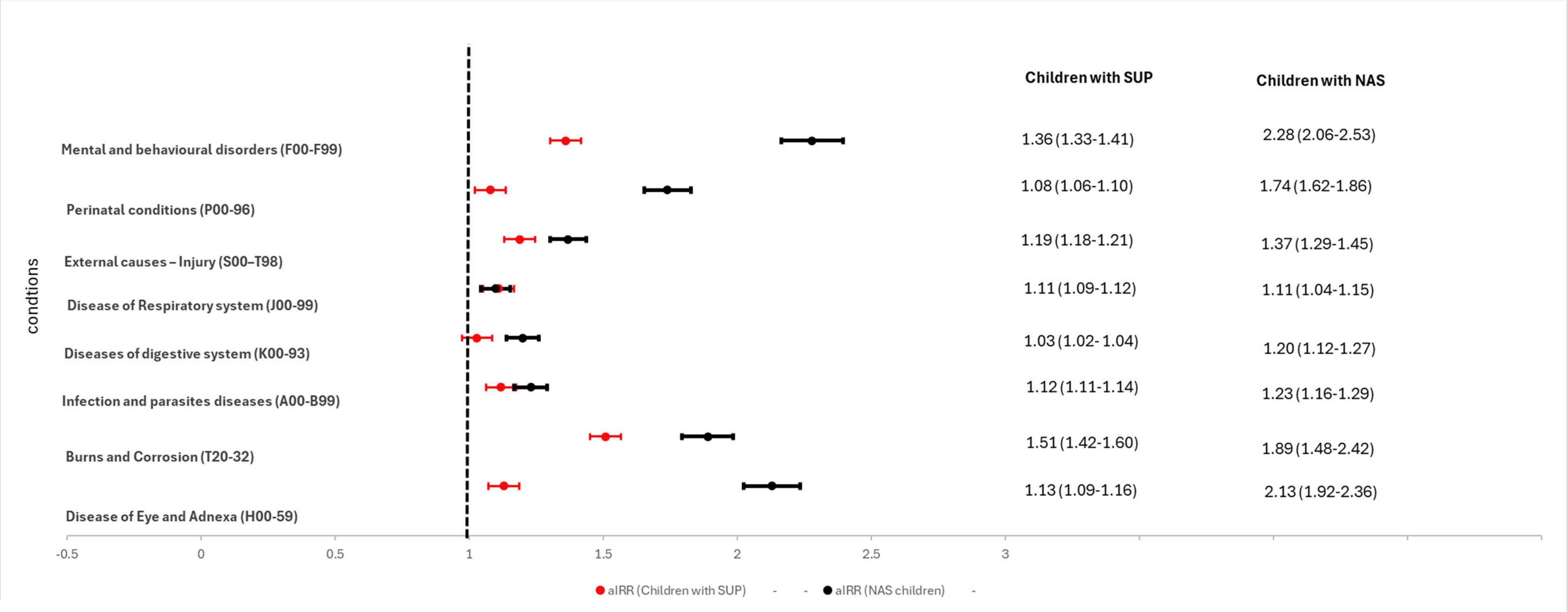

Notes: Relative Risk ratio compared to the children with no known SUP (i.e., children with no known exposure to maternal smoking during pregnancy, maternal alcohol abuse/dependence, maternal drug abuse/dependence, no PDE diagnosis (ICD-10-AM P04.4) or NAS diagnosis (ICD-10-AM P96.1).

Children exposed to SUP = newborns with NAS diagnosis (ICD-10-AM P96.1), PDE diagnosis (ICD-10-AM P04.4, newborn affected by maternal drug of addiction *but no NAS*) or exposed to maternal substance use including alcohol, nicotine, opioids, cannabis, stimulants, sedatives and/or hallucinogens based on ICD-10-AM F10-F19 (*mental or behavioural disorders due to psychoactive substance abuse*) recorded in the mother's last hospital admission or an outpatient mental health care episode within two years before birth.

Children with NAS= newborn identified at birth admission using ICD-10-AM P96.1

Ratios higher than 1.00 indicate that the risk is greater compared with the control group. Ratios in which the 95% CI does not cross 1.00 are statistically significant at the P = .05 level.

Adjusted for maternal age at delivery, young mother aged < 20, self-identified Aboriginal and Torres Strait Islander mothers, index of Relative Socio-economic Advantage and Disadvantage, mother diagnosis of serious mental health disorder based on last hospital admission or episode of mental health care in an ambulatory care setting prior to birth, infant's gender and year of study.

Abbreviation: SUP, Substance use during pregnancy; PDE, Prenatal Drug Exposure; NAS, Neonatal Abstinence Syndrome; ICD-10-AM, International Statistical Classification of Diseases and Related Problems, Tenth Revision, Australian Modification; Adjusted RR, Adjusted Relative Risk; 95%CI, 95% Confidence Interval.

© 2024 Lee E et al. *JAMA Pediatrics.*

eFigure 4: Median time (days) to first re-admission for specific conditions

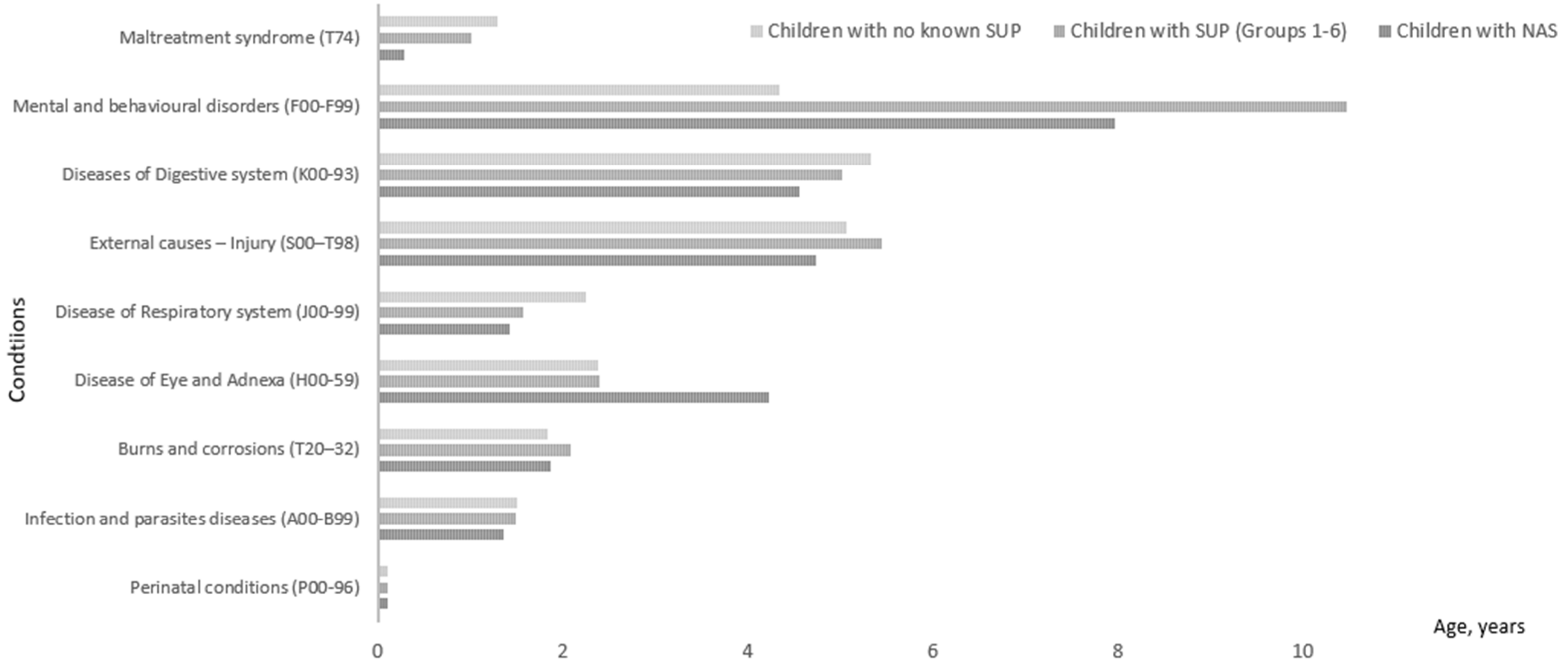

Notes:

Children with <u>no</u> known SUP (i.e., children with no known exposure to maternal smoking during pregnancy, maternal alcohol abuse/dependence, maternal drug abuse/dependence, no PDE diagnosis (ICD-10-AM P04.4) or NAS diagnosis (ICD-10-AM P96.1).

Children exposed to SUP = newborns with NAS diagnosis (ICD-10-AM P96.1), PDE diagnosis (ICD-10-AM P04.4, *newborn affected by maternal drug of addiction but no NAS*) or exposed to maternal substance use including alcohol, nicotine, opioids, cannabis, stimulants, sedatives and/or hallucinogens based on ICD-10-AM F10-F19 (*mental or behavioural disorders due to psychoactive substance abuse*) recorded in the mother's last hospital admission or an outpatient mental health care episode within two years before birth.

Children with NAS= newborn identified at birth admission using ICD-10-AM P96.1

Abbreviations: SUP, Substance use during pregnancy; NAS, Neonatal Abstinence Syndrome; ICD-10-AM, International Statistical Classification of Diseases and Related Problems, Tenth Revision, Australian Modification

© 2024 Lee E et al. *JAMA Pediatrics.*
