# Supplementary material for: Health Care Needs and Costs for Children Exposed to Prenatal Substance Use to Adulthood
Source: JAMA Pediatr. 2024 Jul 22;178(9):888–98. doi: 10.1001/jamapediatrics.2024.2281 (PMC11264092; doi:10.1001/jamapediatrics.2024.2281)
Supplement: Supplement 2. — Data sharing statement [file jamapediatr-e242281-s002.pdf]

Data Sharing Statement

Lee. Health Care Needs and Costs for Children Exposed to Prenatal Substance Use to Adulthood. *JAMA Pediatr*. Published July 22, 2024. doi:10.1001/jamapediatrics.2024.2281

Data
Data available: No

Additional Information
Explanation for why data not available: Data used in these analyses are held by the NSW Ministry of Health. Data are only available on request to the Ministry and access requires appropriate ethical and governance clearances regarding use.
